# Palatal Pedicle Connective Tissue for Reconstruction of Through-and-Through Soft Tissue Defects in Esthetic Zone Around a Dental Implant: An 8-Year Follow-Up Case Report

**DOI:** 10.1155/2024/9936222

**Published:** 2024-10-08

**Authors:** Yuting Wen, Hao Chen, Liping Wang, Shiyong Zhao, Tong Xi

**Affiliations:** ^1^Department of Stomatology, Zigong Fourth People's Hospital, Zigong, China; ^2^Department of Oral and Maxillofacial Surgery, School and Hospital of Stomatology, Guangdong Engineering Research Center of Oral Restoration and Reconstruction & Guangzhou Key Laboratory of Basic and Applied Research of Oral Regenerative Medicine, Guangzhou Medical University, Guangzhou, China; ^3^Department of Oral Implantology, School and Hospital of Stomatology, Guangdong Engineering Research Center of Oral Restoration and Reconstruction & Guangzhou Key Laboratory of Basic and Applied Research of Oral Regenerative Medicine, Guangzhou Medical University, Guangzhou, China 510182; ^4^Department of Oral and Maxillofacial Surgery, Radboud Institute for Health Sciences, Radboud University Medical Center, Nijmegen, Netherlands 6500 HB

**Keywords:** esthetic zone, immediate implant placement, palatal pedicle connective tissue flap, soft tissue augmentation, soft tissue defect

## Abstract

Soft tissue management for immediate implant placement in the esthetic zone is crucial for successful treatment outcomes. Several surgical and restorative techniques have been proposed to treat unesthetic implant soft tissue defects or dehiscence. However, there is a scarcity of clinical evidence on addressing complications associated with through-and-through soft tissue defect (TTSD). In this case, a 61-year-old female patient, who underwent immediate implant placement at the right central incisor 3 months prior, presented with chronic peri-implant mucosal infection and soft tissue recession of TTSD in 2015. The palatal pedicle connective tissue flap (PPCTF) was utilized to provide horizontal and vertical soft tissue augmentation and to cover the defect in combination with the labial contour collapse. At the 8-year follow-up, favorable white and pink esthetic outcomes were observed, and the pink esthetic score (PES) for soft tissue around the implant achieved 9 points, indicating satisfactory esthetic and functional results. The findings from this case study suggest that soft tissue augmentation techniques, such as PPCTF grafts, can provide a reliable and innovative method for both soft tissue augmentation and reconstruction of TTSD, resulting in long-lasting functional and esthetic outcomes and stable ideal PES scores.

## 1. Introduction

Dental implants have emerged as the most promising method of tooth replacement in the modern era. Obtaining a healthy and stable peri-implant soft tissue condition is of significant importance to achieve successful treatment outcomes [1–3]. However, since the implant-soft tissue interface is weakly attached by the epithelial hemidesmosomes and lacks periodontal ligament and cementum, the peri-implant soft tissue is more susceptible to inflammation and infection. Consequently, peri-implant soft tissue is more prone to esthetic complications compared to gingiva around natural teeth [4]. In clinical practice, even with sufficient bone tissue, the alveolar ridge contour may be suboptimal, resulting in peri-implant soft tissue recession, dehiscence, and defects, leading to the exposure of implants or abutments, directly affecting the esthetic outcome of implant restorations. Therefore, it is crucial to apply appropriate methods to enhance the quality and quantity of peri-implant soft tissue, ensuring the long-term survival and success of dental implants.

The complex complications of peri-implant soft tissue have been defined using various terms across studies, including mucogingival defects with bony dehiscence/loss or peri-implant soft tissue dehiscence/defect [5–7]. They described an apical recession on the peri-implant labial soft tissue margin. However, classifications of these complicated conditions such as labial–lingual and multidimensional soft tissue defects combined with labial contour collapse around dental implants have not yet been proposed. This gap in the literature might contribute to complicating clinicians' decision-making when encountering complicated peri-implant soft tissue problems. In this case, we defined a penetrating perisoft tissue defect on the labial–lingual side and coronal of the implant platform as a “through-and-through soft tissue defect” (TTSD), accompanied by labial contour collapse.

Augmentation techniques aiming at enhancing the appearance and dimensions of peri-implant soft tissues are increasingly being utilized to thicken tissue, reestablish adequate keratinized tissue width, correct mucogingival deformities, and improve implant esthetics and stability [8, 9]. Palatal pedicle connective tissue flap (PPCTF) grafts can be the technique of choice in treating peri-implant soft tissue complications [10]. The primary advantages of this flap, harvested from the anterior palate, include primary donor site coverage, increased soft tissue volume at the recipient site, and effective graft integration due to blood supply from both the recipient bed and pedicle base [11, 12]. In the present case, a peri-implant soft tissue presented with TTSD and multidimensional dehiscence was managed with a modified surgical technique, consisting of PPTTF grafting and coverage of the implant site, to repair the peri-implant soft tissue multidimensional TTSD and to reshape gingival contour collapse. Simultaneously, abundant vertical and horizontal soft tissue augmentation in the recipient site was achieved, and the objective clinical outcomes were evaluated by the pink esthetic score (PES). A satisfactory score of 9 has been achieved, resulting in a successful reconstruction of the peri-implant soft tissue and bone tissue stability for up to 8 years.

## 2. Case Presentation

### 2.1. Case Information

**Figure 1:**
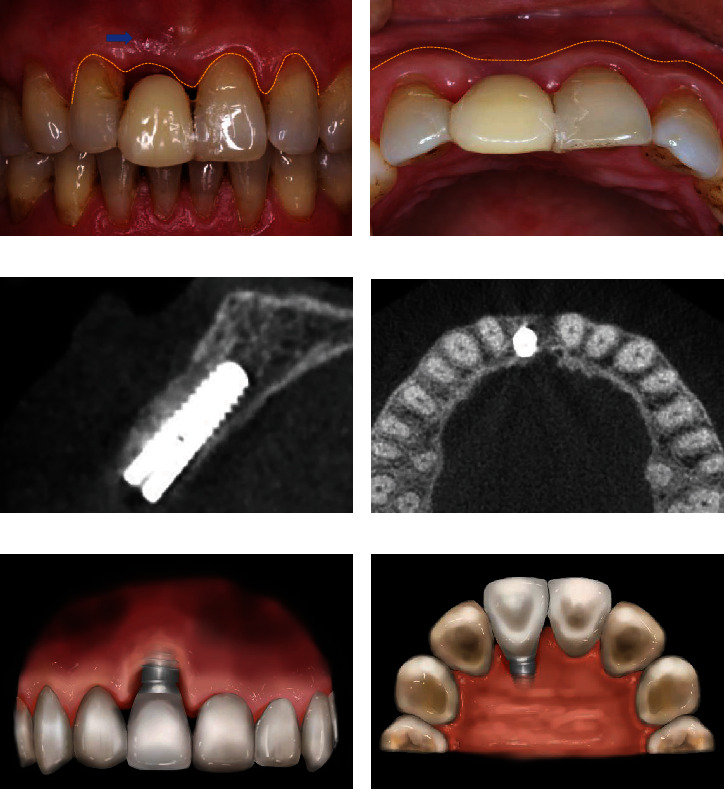
Clinical and radiographic views 3 months postoperative of immediate implant placement. (a) The labial view showing a fistula and papillary recession mesial and distal to the 11. (b) The occlusal view displaying labial peri-implant soft tissue contour collapse. (c, d) The radiographic view 3 months postoperative of implant placement exhibiting no radiolucent lesion around the implant and no resorption of the peri-implant bone. (e) The labial view showed soft tissue recession of TTSD. (f) The lingual view showed soft tissue recession of TTSD.

In 2015, the patient, a 61-year-old female, had undergone immediate implant placement in the #11 (FDI tooth numbering system) 3 months prior. The #11 received a provisional resin restoration. A fistula was observed on the labial side, accompanied by erythema of the marginal gingiva. A peri-implant soft tissue defect manifested with insufficient labial contour and mesial and distal papillary recession of 1.5–2 mm (Figures 1(a) and 1(b)). CBCT examination revealed no radiolucency around the dental implant. The palatal bone plate was located 1.5 mm apical to the implant shoulder. The peri-implant alveolar bone remained intact (Figures 1(c) and 1(d)); no significant resorption of the alveolar process in the labial–palatal dimension occurred.

### 2.2. Treatment Planning

After informing the patient of the clinical condition, the treatment plan was proposed with the patient's informed consent, which involved a surgical debridement of the peri-implant soft tissue followed by a waiting period for the soft tissue inflammation to subside before performing soft tissue augmentation.

### 2.3. Surgical Procedure

**Figure 2:**
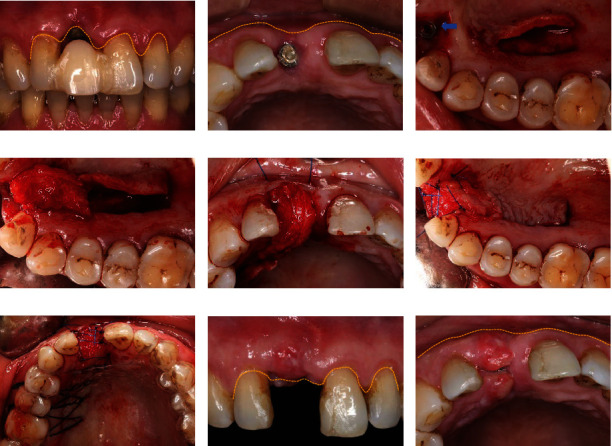
Surgical procedures for transplantation of PPCTF. (a) The labial view of TTSD showing subsided inflammation but horizontally and vertically collapsed peri-implant soft tissue contour. (b) The occlusal view of TTSD revealing recession on the labial–palatal dimension of peri-implant soft tissue and the multidimensional penetrating dehiscence. (c) The peri-implant soft tissue was deepithelialized using a rotating diamond burr (arrow), and incisions on the palatal side were made to raise a partial thickness flap with proper thickness. (d) A PPCTF was elevated to cross the labial–palatal dimension and restore the deepithelialized area around the implant. (e) The PPCTF was mobilized and sutured into the partial thickness flap pocket labial to the implant. (f) Suture fixation around the implant. (g) The vacuum-formed palatal plate with dual functions of wound protection and compression shaping. (h) The labial view 2 weeks postoperative PPCTF transplantation displaying satisfactory horizontal and vertical soft tissue augmentation. (i) The rehabilitation and reconstruction of the labial–palatal dimension of the soft tissue contour. Despite a tiny dehiscence at the crest of the implant, overall peri-implant soft tissue health had been restored.

All surgical operations were performed under infiltration of local anesthesia by articaine 4% (Primacaine Adrenaline, Produits Dentaires Pierre Rolland, France). The steps were as follows:

I. The provisional restoration at #11 was removed, and granulation tissue around the implant was curetted. And the area was rinsed with chlorhexidine. A healing abutment was placed. Three weeks later, during a follow-up visit, the peri-implant soft tissue inflammation had subsided. However, more recession in the labial–palatal dimension of peri-implant soft tissue occurred, causing the alveolar ridge contour to collapse both horizontally and vertically. This resulted in a TTSD with labial contour collapse (Figures 2(a) and 2(b)). The PPCTF was chosen to address the TTSD at #11.
II. Recipient site preparation: The peri-implant soft tissue around the implant was deepithelialized (Figure 2(c)) to prepare for subsequent insertion and fixation of the PPCTF. A partial thickness flap was raised as a pocket on the labial side of the implant.
III. Donor site management: A horizontal incision, extending 3–4 mm straight down to the bone, was made on the palatal side between #13 and #16. At the end of the distal horizontal incision, a vertical incision was made. The full-thickness flap was elevated to determine the thickness of the soft tissue (4–5 mm). A partial thickness flap with appropriate thickness was prepared while keeping the palatal epithelium intact. A second horizontal incision that ran parallel to the first incision was made 10 mm from the first incision line to raise the PPCTF (Figure 2(d)).
IV. Transplantation and fixation: The PPCTF was carefully mobilized to reach the partial thickness flap pocket on the labial side of the implant. After flipping the partial thickness flap, the end of the pedicle flap was sutured with 5-0 polypropylene (Prolene, Ethicon, Johnson & Johnson) into the labial partial thickness flap pocket to repair the TTSD. The PPCTF was also used to bridge the deepithelized tissue between #21 and #12 (Figures 2(e) and 2(f)).
V. Postoperative treatment: A vacuum-formed palatal plate was prepared, which was used to compress the palatal wound, protect the wound at the donor site, and assist in fixating the graft simultaneously. This approach promoted vascularization and graft survival while reducing postoperative pain and swelling (Figure 2(g)). Two weeks later, the wound had healed uneventfully. The peri-implant soft tissue had increased significantly in both horizontal and vertical dimensions. However, due to insufficient vascularization, tiny dehiscence occurred at the crest of soft tissue that covered the healing abutment. This dehiscence resolved gradually upon prosthesis delivery 3 weeks after surgery. The patient was content with the esthetic results (Figures 2(h) and 2(i)).

### 2.4. Prosthesis Delivery and Follow-Up

**Figure 3:**
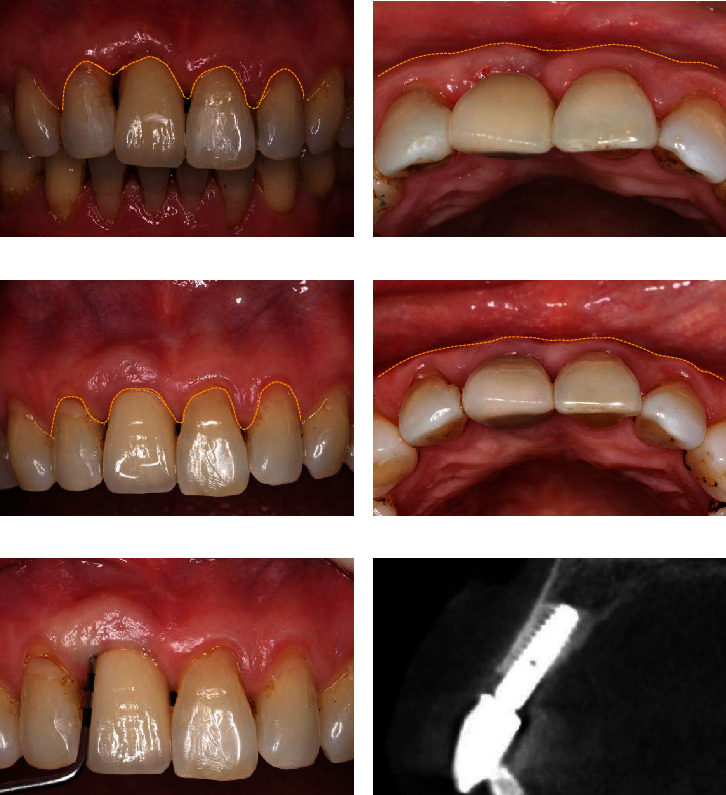
Peri-implant soft tissue condition at the moment of implant crown placement and 8-year follow-up. (a, b) The labial and occlusal view directly after placement of the implant crown, with black triangles visible mesial and distal to the crown. (c, d) The labial and occlusal view at the 8-year follow-up demonstrating a favorable esthetic outcome. (e) A slight overcontouring of the labial soft tissue was present, with no bleeding on probing. (f) The sagittal CBCT image 8 years after implant placement demonstrating a successful osseointegration.

In 2015, an improvement of the peri-implant soft tissue condition was observed after prosthesis delivery, and a satisfactory PES score of 9 points was achieved, indicating the success of the soft tissue augmentation by using a PPCTF (Figures 3(a) and 3(b)). Unfortunately, the patient failed to follow up during this period until 2023, so there were no further records left to supply.

In 2023, the situation remained esthetically and functionally stable 8 years after the implant placement (Figures 3(c) and 3(d)). The labial gingival margin and distal gingival papilla recovered to the same level as the adjacent teeth, with a slight overcontouring of the soft tissue labial to the implant. Clinically, a representative creeping attachment was observed, which resulted in the improved pink esthetics around #11. The labial probing depth of the implant was 5 mm without bleeding on probing (Figure 3(e)). Although there is slight bone resorption of 1–2 mm on the labial side which may be owing to the curettage of unintegrated bone materials during the second stage surgery (Figure 3(f)), the periapical radiograph showed that the peri-implant bone tissue remained relatively and holistically stable for 8 years, and according to the objective clinical PES score, it is still as high as 9 points. Overall, the 8-year follow-up confirmed the long-lasting success of PPCTF in treating TTSD.

## 3. Discussion

Factors that affect postoperative infection in immediate implant placement include chronic infection in the extraction socket, inadequate wound closure, and poor oral hygiene after surgery. Preventive measures include thorough debridement of the infection in the extraction socket, effective wound closure, patient education on postoperative oral hygiene, and appropriate use of antibiotics [13]. In this case, a soft tissue infection occurred during the late healing phase following immediate implant placement. This presented as erythema of the marginal gingiva, swelling, and labial fistula, with an inadequate soft tissue contour and poor esthetic prognosis, but without marginal bone absorption. The diagnosis was peri-implant mucositis, leading to the consideration of debridement and subsequent soft tissue augmentation after inflammation subsided. The cause of the inflammation was speculated to be associated with the unsatisfactory position of the implant, which was close to the adjacent tooth and shallow vertically, as well as plaque accumulation.

The incidence of peri-implant soft tissue defects or dehiscence after immediate implant in the esthetic zone can be as high as 64% [5]. Contributing factors include implant malposition, labial bone fractures or fenestrations, thin gingival biotype, absence or lack of keratinized mucosa, local inflammation, and improper restoration profile [5]. Studies indicated that over one-third of patients undergoing immediate implant placement in the esthetic zone require soft tissue augmentation to achieve optimal outcomes, and the connective tissue graft in maintaining soft and hard tissue volume is necessary [14, 15]. In this case, the patient experienced a collapse of the labial contour and papillary recession 3 months after immediate implantation. Although no bone resorption was present, soft tissue transplantation and augmentation were necessary to achieve ideal and stable esthetic results. However, two major challenges were present: insufficient horizontal and vertical fullness of soft tissue around the implant (TTSD) and unsightly labial contour collapse.

Currently, there are two common soft tissue augmentation approaches, either autogenous or exogenous. Autogenous subepithelial connective tissue grafts, which include free and pedicled subepithelial connective tissue transplantation, are generally considered the gold standard for therapeutic recommendations. However, free connective tissue flaps tend to undergo contraction due to poor blood supply and limited nutrition, which hinders incremental tissue growth. In contrast, PPCTFs have a higher survival rate, as they receive blood supply from their own connective tissue and periosteal vascular plexus, providing predictable incremental effects and a sound biological basis. All of this prevents graft necrosis in complex soft tissue defect situations [10, 16].

Additionally, PPCTFs offer good vascularization, reduced contraction, and easier intraoperative fixation. Some recent studies have proposed classification systems for peri-implant soft tissue dehiscence/deficiency (PSTD) and marginal mucosa defects, along with possible treatment strategies for each condition [6, 7, 17]. However, it is important to note that this classification and decision-making process lacks a further category and recommended surgical approaches for TTSD along with labial contour collapse. As a result, a more suitable and innovative soft tissue augmentation method was necessary to address these issues.

In this study, we present a case that highlights innovative techniques and advantages of using a PPCTF to treat vertical and horizontal soft tissue insufficiencies of TTSD. Our findings are as follows:

I. The PPCTF could provide a rich blood supply and a high survival rate. It mainly consists of lamina propria and has a low proportion of submucosa, making it well-vascularized and significantly enhancing the thickness of the soft tissue in the recipient area. This technique significantly increases both horizontal and vertical soft tissue thickness, ensuring proper sealing of the implant platform and facilitating graft wound healing. The outcome is stable and esthetically pleasing.
II. We prepared a partial thickness flap that was shaped as a pocket on the labial side of the implant in the recipient area, providing a blood supply and nutrient-rich implantation bed for the flap graft. The graft was secured in the recipient site using suture fixation mode to achieve a pocket-seal effect, which is conducive to the formation and vascularization of soft tissue augmentation [18].
III. The study highlighted the importance of using a vacuum-formed palatal plate to compress the wound of the donor and recipient areas, reducing postoperative morbidity and maintaining the incremental shape of the soft tissue in the early stage of healing. This stimulated vascularization through moderate compression.
IV. From a postoperative healing perspective, a small dehiscence of the PPCTF was observed above the healing abutment 2 weeks after surgery. However, the blood supply of the soft tissue in the labial and palatal regions had already been established, and the soft tissue volume increased significantly after the operation. The side defect was significantly improved, and the ideal incremental effect was achieved in the vertical and horizontal soft tissue deficiencies. The elasticity and color of the palatal mucosa flap adjacent to the gingival papilla laid the foundation for the favorable soft tissue esthetics in the 8-year follow-up observation.
V. This case provides a new clinical decision-making idea for vertical and horizontal soft tissue insufficiency combined with TTSD and labial contour collapse.

In the 8-year follow-up, the soft tissue surrounding the implant appeared overcontoured compared to the normally attached gingiva of adjacent teeth, with the gingival papilla being satisfactorily filled and bone stability around the implant deemed acceptable. Studies have shown that increases in fibrosis are key factors contributing to hypertrophic scarring in the operation area. According to the theory of cell subpopulation [19], it is inferred that the subpopulation of gingival fibroblasts in the palatal donor site in this case is heterogeneous and has different phenotypes [20, 21]. The varied response of gingival fibroblasts to different stimuli is related to collagen metabolism, which leads to the partial occurrence of overcontoured soft tissue appearance in the labial of dental implants. Moreover, the abundance of volume and thickness of soft tissue stimulates and induces peri-implant soft tissue “creeping attachment” [19] with the adjacent teeth, creating an impressive esthetic regeneration over 8 years. Despite not undergoing bone augmentation surgery, the clinical objective PES scores were still maintained.

## 4. Conclusion

This study illuminated that the PPCTF can serve as an effective technique for the reconstruction of TTSD in conjunction with labial contour collapse. The 8-year follow-up indicated that soft tissue augmentation using PPCTF could result in stable peri-implant soft tissues. This innovative approach has the potential to promote graft vascularization and enhance the augmentation effects in peri-implant complex complications. Further research is necessary to understand more detailed techniques for optimizing esthetic, functional, and biological outcomes of TTSD and to provide significant evidence-based proof with reproducibility and practicality for clinical decision-making.

## Data Availability

The complete data and materials described in the case report are freely available from the corresponding author on reasonable request.
